# The impact of substance misuse in patients with first-episode psychosis: a retrospective study

**DOI:** 10.1192/j.eurpsy.2023.2300

**Published:** 2023-07-19

**Authors:** R. A. Moreira, H. J. Gomes, J. P. Correia, E. Maldonado, J. M. Justo

**Affiliations:** Departamento de Psiquiatria e Saúde Mental, Unidade Local de Saúde do Nordeste, Bragança, Portugal

## Abstract

**Introduction:**

Substance misuse increases the risk of developing psychosis in vulnerable people. However, patients with substance use disorders’ attributes and their effect on first-episode psychosis (FEP) are still unclear.

**Objectives:**

To describe and compare inpatient admissions for FEP with substance misuse and its impact on clinical outcomes.

**Methods:**

We conducted an observational and retrospective study, analyzing sociodemographic determinants and clinical data regarding the patients hospitalized for FEP, between January 2019 and June 2022, in the psychiatric unit at our hospital in Bragança, Portugal. We used logistic regression to estimate the effect of social determinants and other clinical data regarding the patients hospitalized for FEP with substance abuse.

**Results:**

We included 78 patients in this study. Of these patients, 30% (n=23) reported substance (drugs or alcohol) misuse prior to hospital admission. Regarding only the patients with substance misuse, 96% were male and the median age was 31 years. Cannabis was the most often reported substance of abuse (83%). Most of these patients were unmarried (OR:10.794; 95%CI:2.855-40.805;P=0.001), lived in a rural setting (OR:0.263; 95%CI:0.094-0.731;P=0.009) and had no previous psychiatric history (OR:1.022; 95%CI:0.386-2.709;P=0.964).Regarding hospital admission, 70% were involuntary admitted (OR:4;95%CI:1.408-11.366;P=0.007) and the median time of hospitalization was 17 days. At the time of discharge, 48% of these set of patients still didn’t have insight into their mental illness (OR:1.737;95%CI:0.646-4.679;P=0.272). During the evaluation period of this study, 13% of the patients were readmitted to the hospital (OR:1.029;95%CI:0.241-4.383;P=0.970) and 35% missed outpatient appointments (OR:3.133;95%CI:1.003-9.791;P=0.044). The diagnoses at the time of discharge were: substance-induced psychosis (52%), schizophrenia (22%), affective psychosis (17%), and acute and transient psychotic disorder (9%).

**Conclusions:**

This analysis indicates substance misuse predates and is prevalent in FEP. Many of these patients fail to recognize and accept that they are suffering from a mental illness and drop out of outpatient psychiatric care. Further, substance-induced psychoses are associated with a significant risk for transition to schizophrenia particularly following cannabis-induced psychosis. Thus, it is crucial to optimize adherence to the therapeutic regimen and outpatient follow-up.

**Disclosure of Interest:**

None Declared

